# Nutritional status of flexitarians compared to vegans and omnivores - a cross-sectional pilot study

**DOI:** 10.1186/s40795-023-00799-6

**Published:** 2023-11-28

**Authors:** Anja Bruns, Josefine Nebl, Wiebke Jonas, Andreas Hahn, Jan Philipp Schuchardt

**Affiliations:** Institute of Food Science and Human Nutrition, Leibniz University Hannover, Hannover, 30167 Germany

**Keywords:** Flexitarians, Nutritional status, Critical parameters, Vegans, Plant-based diet, Cobalamin, Vitamin D, Iron, Supplementation

## Abstract

**Background:**

In the Western world, there has been a notable rise in the popularity of plant-based, meat-reduced flexitarian diets. Nevertheless, there is insufficient data on the nutritional status of individuals following this dietary pattern. The aim of this study was to investigate the intake and endogenous status of various nutrients in a healthy German adult study population consisting of flexitarians (FXs), vegans (Vs) and omnivores (OMNs).

**Methods:**

In this cross-sectional study, dietary intake of 94 non-smoking adults (32 FXs, 33 Vs, 29 OMNs) between 25 and 45 years of age was assessed using 3-day dietary records. In addition, blood samples were collected to determine different endogenous nutrient status markers.

**Results:**

32%, 82% and 24% of the FXs, Vs, and OMNs respectively reported using dietary supplements. In the FXs, intake of total energy as well as macronutrients and most micronutrients were within the reference range. FXs had higher intakes of fiber, retinol-equ., ascorbic acid, folate-equ., tocopherol-equ., calcium, and magnesium compared to OMNs. However, cobalamin intake in FXs (2.12 µg/d) was below the reference (4 µg/d). Based on 4cB12, 13% of FXs showed a cobalamin undersupply [< -0.5 to -2.5] compared to 10% of OMNs, and 9% of Vs. The median 25(OH)D serum concentrations in FXs, Vs and OMNs were 46.6, 55.6, and 59.6 nmol/L. The prevalence of an insufficient/deficient vitamin-D status [< 49.9 nmol 25(OH)D/L] was highest in FXs (53%), followed by Vs (34%) and OMNs (27%). In FXs and Vs, the supplement takers had better cobalamin and vitamin-D status than non-supplement takers. Anemia and depleted iron stores were found only occasionally in all groups. In women, the prevalence of pre-latent iron deficiency and iron deficiency was highest in FXs (67%) compared to Vs (61%) and OMNs (54%).

**Conclusion:**

Our findings indicated that all three diets delivered sufficient amounts of most macro- and micronutrients. However, deficiencies in cobalamin, vitamin-D, and iron status were common across all diets. Further studies are needed to investigate the nutrient supply status and health consequences of meat-reduced plant-based diets. The study was registered in the German Clinical Trial Register (number: DRKS 00019887, data: 08.01.2020).

**Supplementary Information:**

The online version contains supplementary material available at 10.1186/s40795-023-00799-6.

## Introduction

In recent years, plant-based diets have gained interest for a variety of reasons, including ecological, ethical and health considerations, and the number of people following these diets in the Western world has increased. Additionally to vegetarianism and veganism, a plant-based, meat-reduced flexitarian (FX) dietary pattern, characterised by a consciously reduced, “flexible” consumption of meat and meat products, is also gaining popularity [1–4]. However, up to now the term “flexitarianism” has been interpreted in different ways. Springmann and colleagues [5] define the consumption of meat and meat products ≤ 1 time per week as a FX diet, while Papier et al. [6] suggest a consumption of < 2–3 times/week meat and meat products as a FX diet. Dagevos [7] identifies a FX diet when meat is eaten occasionally without avoiding it entirely. In the present study, participants were defined as FXs if they consumed ≤ 50 g/day (equivalent to ≤ 350 g/week) of meat and meat products, which reflects the lower limits of recommendations from several different nutrition societies [8–11].

When evaluating a diet, consideration of nutrient intake and endogenous status always plays an important role. It is undisputed that a lacto-ovo-vegetarian diet, based on a wide range of foods, generally fulfils all nutritional requirements in generally in adults [12–14]. In contrast, a strict vegan diet can be deficient with respect to micronutrients such as cobalamin as well as calcium, iron and zinc [15–17]. Despite growing interest, data on the nutritional situation of FX diets are limited. For example, recent studies by Kwasniewska et al. (2023) and Dawczynski et al. (2022) compared the nutritional status of different plant-based diets (including FXs) with an omnivorous dietary pattern [18, 19]. Their results showed that the intake of several micronutrients can be deficient in FXs. Overall, however, there is currently very little data on the supply of FXs with (critical) nutrients. Groufh-Jacobsen et al. [20] found that young FXs and vegans had higher general nutritional knowledge than lacto-ovo vegetarians, pescatarians and omnivores, although food literacy was moderate across all dietary practices.

Thus, the aim of this cross-sectional pilot study was to compare the nutrient intakes of healthy young and middle-aged subjects on a FX diet with those of vegans and omnivores and evaluate the actual intakes in relation to recommended intakes. To identify potential deficiencies, endogenous concentrations of cobalamin, folate, 25(OH)D, iron and related biomarkers were monitored and evaluated. In addition, differences in concentrations of nutrient status markers depending on the intake of supplements were examined.

## Methods

### Study design and participants

The cross-sectional study was designed and conducted at the Institute of Food Science and Human Nutrition of Leibniz University Hannover, Germany, (hereafter referred to as the Institute) according to the guidelines of the Declaration of Helsinki. It was approved by the Ethics Committee of the Medical Association of Lower Saxony in Hannover, Germany, on the 9th of September 2019 (43/2019). All subjects gave their written informed consent for the use of the data collected prior to their participation. The study was registered in the German Clinical Trial Register under the number DRKS 00019887 (registration data: 08.01.2020). In addition, the STROBE guidelines were applied [21].

The entire study design was published recently [22]. Nutrient intake and status of selected nutrients were compared among FXs, vegans and omnivores. Interested persons were included in the study if they followed the diet for ≥ 1 year:

- flexitarian (FX) diet, if they consumed meat and meat products ≤ 50 g/day (equivalent to ≤ 350 g/week)
- vegan (V) diet, if they consumed no food of animal origin
- omnivore (OMN) diet, if they consumed meat and meat products ≥ 170 g/d on average (equivalent to ≥ 1190 g/week)

Meat and meat products consumption was defined as follows: meat = red and white meat, meat products = ham, sausage, cold cuts, meatballs, meat nuggets.

Consumption limits for meat and meat products were based on [8, 10, 11] for FXs and on per capita consumption between 2011–2018 for OMNs in Germany and on the European average, respectively [23, 24]. Subjects with a consumption of meat and meat products ≥ 50 g/d ≤ 170 g/d were not included in order to achieve a clear differentiation between FXs and OMNs.

We assessed the eligibility of potential participants in several steps. First, subjects had to fulfil an online screening questionnaire. The online screening questionnaire mainly contained questions about the in- and/or exclusion criteria (e.g. age, sex, anthropometrics, health status), but also specific questions about the diet (in particular the consumption of meat and meat products, milk and milk products, eggs etc.) and how long they had been following the diet to check whether the people were suitable for the FX and OMN or the V group. Secondly, potentially suitable people were invited for a face-to-face interview. The subjects were interviewed about the amount of meat and meat products consumed. After the interview it was decided whether the subjects could be included in the study.

The aim was to create a homogeneous cohort in a narrow age range. Hence, people in the age range between 25 and 45 years were included in the study. There were two main reasons for choosing this age group: First, people who follow an FX diet are most likely to be found in this age range. Second, a high level of adherence and motivation can be expected from subjects in this age group.

**Fig. 1:**
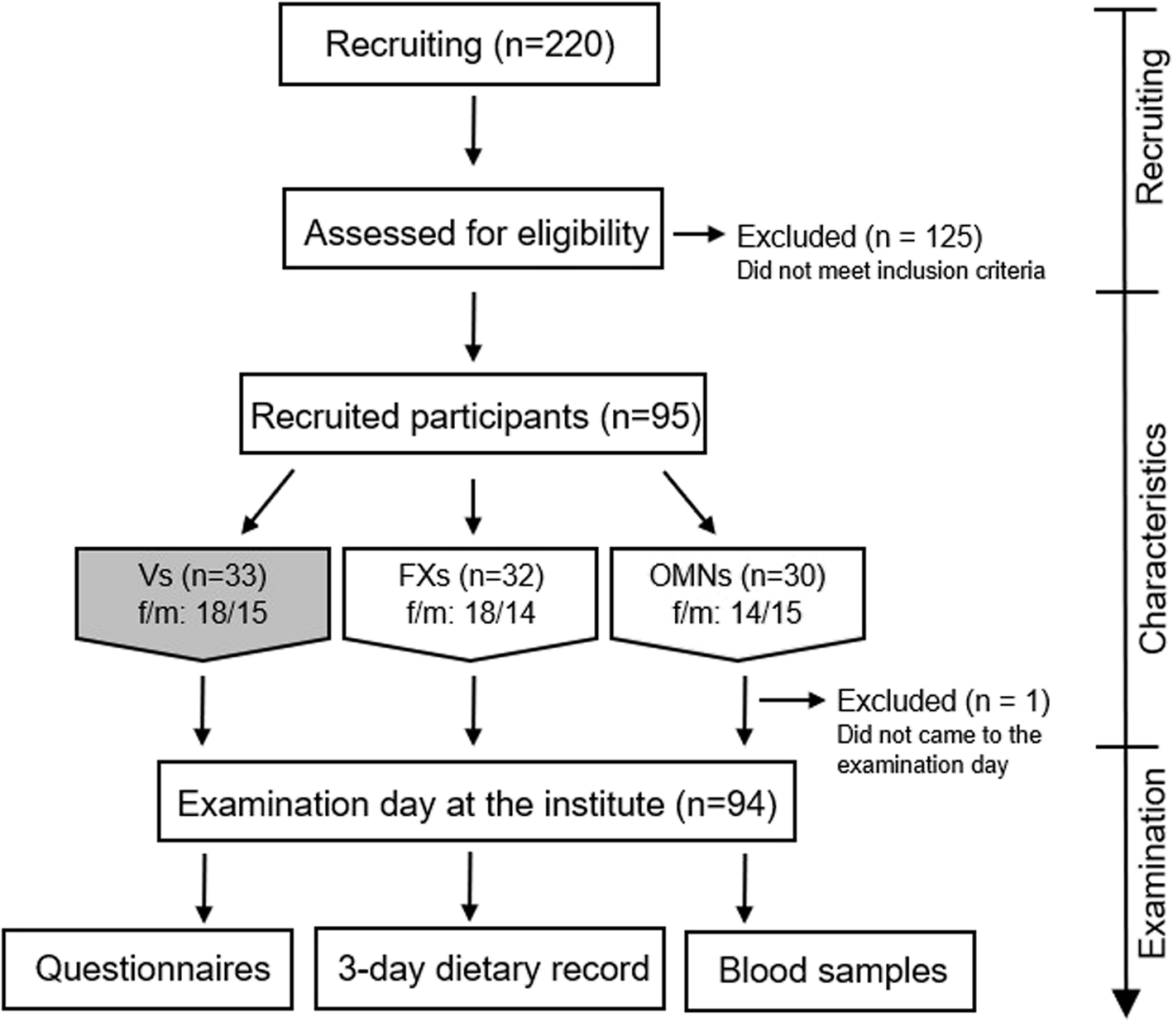
Flow chart of the study

Further inclusion criteria for participation were: body mass index (BMI) between 20 and 28 kg/m^2^, metabolically healthy and non-smoker. In contrast, acute febrile infections, metabolic or malignant diseases, diseases of the gastrointestinal tract, pregnancy or lactation, endocrine and immunologic diseases, food intolerances and drug or alcohol dependence led to exclusion. Recruited subjects were matched for age and sex within each group and across the three groups (Fig. 1). Finally, eligible participants were invited to attend an examination day at the Institute. The study was carried out between March and August 2020.

### Dietary records and questionnaire of physical activity

Prior to the examination day, participants self-recorded their dietary habits using 3-day dietary records for three consecutive days, including one weekend day. All food quantities were estimated by the participants in household measures or grams. In order to increase the accuracy, participants were instructed in how to complete the 3-day dietary records in face-to-face interview prior study begin. In addition, the blank 3-day food diaries contained detailed information on examples of portion sizes, of sharing recipes or weighing food, etc. All food records were checked for completeness, legibility, and plausibility by nutritionists of the Institute. Any discrepancies were resolved by the dietitians personally with the study participants. In addition, the participants were asked whether they used dietary supplements (yes/no). In general, intakes without supplements were reported. The validated Freiburg questionnaire was used to assess health-related physical activity [25].

### Anthropometric data

On the day of the examination at the Institute, height was measured with a stadiometer (Seca GmbH & Co. KG, Hamburg, Germany). Body weight was determined digitally (Seca GmbH & Co. KG, Hamburg, Germany) to the nearest 0.1 kg (lightly dressed, without shoes). From these data, the BMI was calculated according to the standard formula, i.e., the ratio of weight and height to the square [26]. All measurements were taken by trained nutritionists of the Institute.

### Biochemical markers

Blood samples were collected by a licensed physician after an overnight fast (≥ 12 h) between 06:00 a.m. and 11:00 a.m. Samples were obtained by puncture of an arm vein with multifly needles into serum monovettes (3 × 7.5 ml), serum gel monovettes (1 × 7.5 ml), and EDTA monovettes (2 × 2.5 ml) from Sarstedt, Nümbrecht, Germany. A 2.5 ml EDTA monovette was centrifuged to separate plasma from serum (10 min at 2500 g). All samples were stored below 5 °C and transported to the laboratory on the same day. The analysis of all blood parameters was performed in an accredited and certified laboratory (Institute of Clinical Chemistry, Hannover Medical School, Germany).

The cobalamin status was evaluated by serum cobalamin, holotranscobalamin (Holo-TC), methylmalonic acid (MMA), total homocysteine (tHcy), and the cobalamin indicator 4cB_12_. Cobalamin in serum as well as parathormone (PTH) were determined by electrochemiluminescence immunoassay (ECLIA) on Cobas® 8000, module e801, Roche Diagnostics GmbH, Mannheim, Germany. Enzyme immunoassay (ELISA) was used for Holo-TC in serum from Tecan Trading AG, Hamburg, Germany. Gas chromatography coupled to mass spectrometry (GC-MS/MS) was used to analyse MMA in serum (Agilent Technologies GmbH, Waldbronn, Germany). tHcy was determined using the Cobas® 8000, module c502, Roche diagnostics GmbH, Mannheim, Germany, enzyme cycle assay. The folate status was evaluated by serum folate concentrations, which were analyzed by ECLIA on the Cobas® 8000, module e801, Roche diagnostics GmbH, Mannheim, Germany. To calculate the cobalamin indicator 4cB_12_, four markers were calculated according to the following formula [27]:

$$4cB12=\mathrm{log}10 (\frac{HoloTC*B12}{MMA*tHcy})-age\, factor$$

To evaluate the vitamin D status, serum 25-hydroxyvitamin D [25(OH)D] was analyzed using chemiluminescence immunoassay (CLIA) from Liaison XL, DiaSorin GmbH, Dietzenbach, Germany.

Serum iron was assessed using a spectrophotometric method on the Cobas 8000 module c701 (Roche Diagnostics GmbH, Mannheim, Germany). Serum ferritin was measured by ECLIA and serum transferrin by immunoturbidimetric assay using Cobas 8000, module c701 (Roche Diagnostics GmbH, Mannheim, Germany). Haemoglobin (Hb) was determined by capillary electrophoresis (Sebia GmbH, Fulda, Germany). Transferrin saturation, haematocrit (Hct) and mean corpuscular volume (MCV) were calculated using standard formulas.

### Reference values

The Dietary Reference Intakes (DRI) values of the German, Austrian and Swiss Nutrition Societies (D-A-CH) [28] and European Food Safety Authority (EFSA) [29] were used to evaluate nutrient intakes.

Cut-offs for cobalamin and cobalamin status-related biomarkers were applied as follows: Serum cobalamin < 150 pmol/L (deficient cobalamin status) [30], MMA > 271 mmol/L (elevated) [31, 32], Holo-TC < 35 pmol/L (deficient cobalamin status) [33], tHcy > 10 µmol/L (elevated) [34, 35]. The calculated values of the 4cB_12_ marker were classified into five age-adjusted categories: probable cobalamin deficiency (< -2.5), possible cobalamin deficiency (-2.5 to -1.5), low cobalamin (-1.5 to -0.5), cobalamin adequacy (-0.5 to 1.5), elevated cobalamin (> 1.5) [27].

In accordance with the recommendations from the Institute of Medicine (IOM) and the D-A-CH Nutrition Society, the cut-off for serum 25(OH)D concentrations was set at > 50 nmol/L as an indicator of adequate vitamin D status [36–40]. 25(OH)D concentrations > 75 nmol/L were considered as desirable in view of bone health [41]. Further cut-offs were drawn, in order to evaluate concentrations below 50 nmol/L: 25(OH)D concentrations between 25–< 50 nmol/L were classified as “insufficient” and concentrations < 25 nmol/L as “deficient” according to the classification of numerous recent publications [36–38].

The WHO (2015) guidelines were consulted to determine serum folate concentrations (deficient < 6.8 nmol/L) [42]. Parameters of the iron status (serum iron: pre-latent iron deficiency (< 20-14 µmol/L), deficient (< 14 µmol/L); ferritin: depleted iron stores (< 15 µg/l); transferrin saturation: Insufficient iron supply (< 16%); Hb: Anaemia (female < 12 g/dl, male < 13 g/dl); Hct: Deficiency (female < 36%, male < 39%; MCV: Iron deficiency anaemia (< 80 fl)) were set according to WHO [43].

### Data analysis and statistical methods

The sample size of *n* = 25 per group was based on a significance level (alpha) of 0.05 and a beta of 0.8 to detect between-group differences, assuming an effect size ≥ 0.8. A minimum of 30 participants per intervention group were enrolled, considering an expected dropout rate of 15%. Statistical analyses were performed using SPSS software IBM SPSS Inc. Statistics 27.0.1.0, Chicago, Il, USA. The normal distribution of the data was tested using the Kolmogorov-Smirnov test. If the data were normally distributed, one-way analysis of variance (ANOVA) was used. If the data were not normally distributed, the Kruskal-Wallis test was used. The post hoc test with Bonferroni correction was applied for significant differences, and the Chi-square test was used to compare frequencies between the three diets. Differences between intake and reference values (100%) were determined using the one-sample t-test for normally distributed data and the Wilcoxon test for non-normally distributed data. Calculations of daily intakes of macro- and micronutrients were performed using the nutrition software PRODI 8.11 (Nutri-Science GmbH, Freiburg, Germany). Statistical significance was set up at *p*-values ≤ 0.05. Results are presented as median ($\widetilde{x}$) with interquartile range (25^th^ and 75^th^ percentiles).

## Results

### Characterisation of the study population

A total of 94 eligible young/middle-aged subjects participated in the present study. 32 were FXs, 33 were Vs, and 29 were OMNs (Table 1). Within each group and across all three diets, sex and age were matched. However, there were differences in the duration of the diets in the different groups: OMNs maintained their diet significantly longer than Vs and FXs. FXs and Vs showed higher rates of physical activity (h/week), although the difference between Vs and OMNs was significant. In all groups, the BMI was within the normal weight range, with FXs having significantly lower values than OMNs (*p* = 0.003). 32% of FXs reported taking dietary supplements, compared to 82% of Vs and 24% of OMNs. Thus, FXs and OMNs were significantly less likely to use dietary supplements (p ≤ 0.001 respectively).

**Table 1:** Characterisation of the study population

**Parameter**	**FX**	***P*** **FX-V**	**V**	***P*** **V-OMN**	**OMN**	***P*** **FX-OMN**	***P*** **overall**
	${\varvec{n}}/\widetilde{{\varvec{x}}}({\varvec{I}}{\varvec{Q}}{\varvec{R}})$		${\varvec{n}}/\widetilde{{\varvec{x}}}({\varvec{I}}{\varvec{Q}}{\varvec{R}})$		${\varvec{n}}/\widetilde{{\varvec{x}}}({\varvec{I}}{\varvec{Q}}{\varvec{R}})$		
Total Participants (n) (female/male)	32 (18/14)	n.s	33 (18/15)	n.s	29 (13/16)	n.s	0.633^a^
Age (years)	32 (26–36)	n.s	33 (29–37)	n.s	32 (28–43)	n.s	0.377^b^
BMI (kg/m^2^)	22.0 (21.0–25.0)	0.375	23.0 (22.0–25.0)	0.223	25.0 (23.0–27.0)	**0.003**	**0.005**^**b**^
Duration of diet		**≤ 0.001**		**≤ 0.001**		**≤ 0.001**	**≤ 0.001**^a^
5 years, n	19		22		1		
6–10 years, n	6		9		1		
11 years, n	7		2		27		
**Lifestyle factors**							
Physical activity (h/week)	10.2 (7.70–14.7)	0.958	12.0 (7.50–16.1)	**0.007**	7.80 (4.00–9.30)	0.106	**0.008**^c^
Supplement-User (n) (%)	10 (32)	**≤ 0.001**	27 (82)	**≤ 0.001**	7 (24)	1.000	**≤ 0.001**^a^

*P* ≤ 0.05 was considered significant

*P*-values in bold represent statistical significance

*IQR* Interquartile range, *FXs* Flexitarians, *Vs* Vegans, *OMNs* Omnivores

Differences between groups were analysed using either:

^a^Chi-Square-Test, ^b^Kruskal-Wallis test with post hoc test and Bonferroni correction, ^c^One-Way ANOVA

### Nutrient intake

#### Total energy, macronutrient, dietary fiber and alcohol intake

The recommended intakes for total energy were met by FXs, whereas Vs and OMNs were below the recommended intakes (Table 2). No group differences were found. Vs had the highest carbohydrate intakes and reached reference values, whereas FXs and OMNs were below the recommended intakes. Fat intake was highest in OMNs, who exceeded the recommended intakes, followed by FXs, who were close to the recommended values. Vs had fat intakes within the reference corridor. As expected, protein intake was highest in the OMNs, followed by the FXs and the Vs. All three diets met the population reference intakes in g/kg body weight/day for protein, although the EN% recommendations were not met, mainly because of the high fat intakes. Fiber intakes were significantly different between the three groups, with Vs having the highest intakes, followed by FXs and OMNs. Reference intakes for fiber were met only by FXs and Vs. Irrespective of gender, all diets were below the maximum tolerated alcohol intake.

**Table 2:** Total energy, macronutrient, dietary fiber and alcohol intake

**Parameter **[unit/day]	**FX**	***P*** **FX-V**	**V**	***P*** **V-OMN**	**OMN**	***P*** **FX-OMN**	***P*** **overall**	**Recommendations**	
								**D-A-CH**^**c**^ [28]	**EFSA**^**d**^ [29]
	$\widetilde{{\varvec{x}}}({\varvec{I}}{\varvec{Q}}{\varvec{R}})$		$\widetilde{{\varvec{x}}}({\varvec{I}}{\varvec{Q}}{\varvec{R}})$		$\widetilde{{\varvec{x}}}({\varvec{I}}{\varvec{Q}}{\varvec{R}})$				
Total Energy [kcal]^f^									
Total	2160 (1885–2672)	n.s	2063 (1654–2488)	n.s	2064 (1877–2349)	n.s	0.478^a^	n.s	n.s
f	2098 (1884–2197)	n.s	1979 (1599–2318)	n.s	1935 (1877–2107)	n.s	0.772^a^	2100	2078
m	2598 (2292–2919)	n.s	2127 (1886–2918)	n.s	2323 (1816–2759)	n.s	0.333^a^	2700	2579
Carbohydrates [EN%]									
Total	43.4 (39.0–48.3)	n.s	47.0 (40.3–51.7)	n.s	38.9 (34.4–41.9)	n.s	0.463^a^	≥ 50	45–60
f	43.9 (37.8–49.8)	0.899	42.4 (35.5–50.9)	**0.024**	37.7 (34.4–42.4)	0.301	**0.028**^**b**^	n.s	n.s
m	43.4 (42.0–46.8)	0.279	49.1 (42.5–52.6)	**0.013**	39.9 (35.5–41.9)	0.771	**0.016**^**a**^	n.s	n.s
Fat [EN%]									
Total	36.2 (32.0–40.3)	n.s	32.8 (27.5–42.2)	n.s	40.4 (34.7–42.6)	n.s	0.805^b^	30	20–35
f	35.8 (31.0–39.0)	n.s	36.2 (30.2–47.2)	n.s	40.7 (32.3–43.0)	n.s	0.278^b^	n.s	n.s
m	38.8 (32.5–40.7)	0.096	30.9 (25.7–37.2)	**0.027**	39.9 (37.5–42.0)	1.000	**0.021**^**b**^	n.s	n.s
Protein [EN%]									
Total	14.5 (12,8–16.1)	0.237	13.4 (11.7–14.6)	**0.001**	16.1 (14.2–18.7)	0.135	**0.001**^**a**^	20	n.s
f	14.2 (13.3–15.9)	0.287	12.5 (11.6–14.2)	**≤ 0.001**	16.5 (15.2–20.7)	0.072	**0.001**^**a**^	n.s	n.s
m	15.8 (12.8–16.3)	n.s	13.8 (12.5–16.2)	n.s	15.8 (13.8–17.7)	n.s	0.371^b^	n.s	n.s
Protein [g/kg body weight]									
Total	1.11 (0.98–1.37)	n.s	0.97 (0.74–1.29)	n.s	1.20 (0.98–1.43)	n.s	0.104^a^	n.s	0.83^e^
f	1.11 (0.96–1.33)	0.371	0.96 (0.77–1.17)	**0.032**	1.20 (1.08–1.44)	0.754	**0.035**^**a**^	n.s	n.s
m	1.12 (1.02–1.54)	n.s	1.02 (0.66–1.49)	n.s	1.13 (0.87–1.40)	n.s	0.398^b^	n.s	n.s
Dietary fiber [g]									
Total	28.9 (23.5–37.6)	**0.013**	40.6 (30.9–52.8)	**≤ 0.001**	18.8 (16.7–25.4)	**≤ 0.001**	**0.001**^**a**^	30	25
f	27.2 (23.2–30.9)	0.114	38.1 (28.3–50.3)	**≤ 0.001**	19.5 (18.7–22.9)	**0.050**	**0.001**^**a**^	n.s	n.s
m	34.6 (27.6–39.2)	0.272	42.0 (35.3–59.5)	**≤ 0.001**	18.0 (13.2–25.6)	**0.004**	**0.001**^**a**^	n.s	n.s
Alcohol [g]									
Total	4.58 (0.18–9.07)	**0.033**	0.00 (0.00–0.73)	0.054	3.12 (0.00–13.1)	1.000	**0.016**^**a**^	n.s	n.s
f	2.84 (0.18–9.70)	n.s	0.03 (0.00–0.55)	n.s	0.17 (0.00–3.12)	n.s	0.052^a^	10	n.s
m	5.68 (0.36–8.92)	n.s	0.00 (0.00–12.6)	n.s	7.58 (1.80–15.9)	n.s	0.064^a^	20	n.s

*P* ≤ 0.05 was considered significant

Values in bold represent statistical significance

*IQR* Interquartile range, *EN%* Percentage of energy intake, *FXs* Flexitarians, *Vs* Vegans, *OMNs* Omnivores

^a^Kruskal-Wallis test with post hoc test and Bonferroni correction, ^b^One-Way-Anova

^c^Reference values

^d^Average requirement

^e^Population reference intake

^f^PAL = 1.6 and aged between 25-51 years

#### Micronutrient intake

**Fig. 2:**
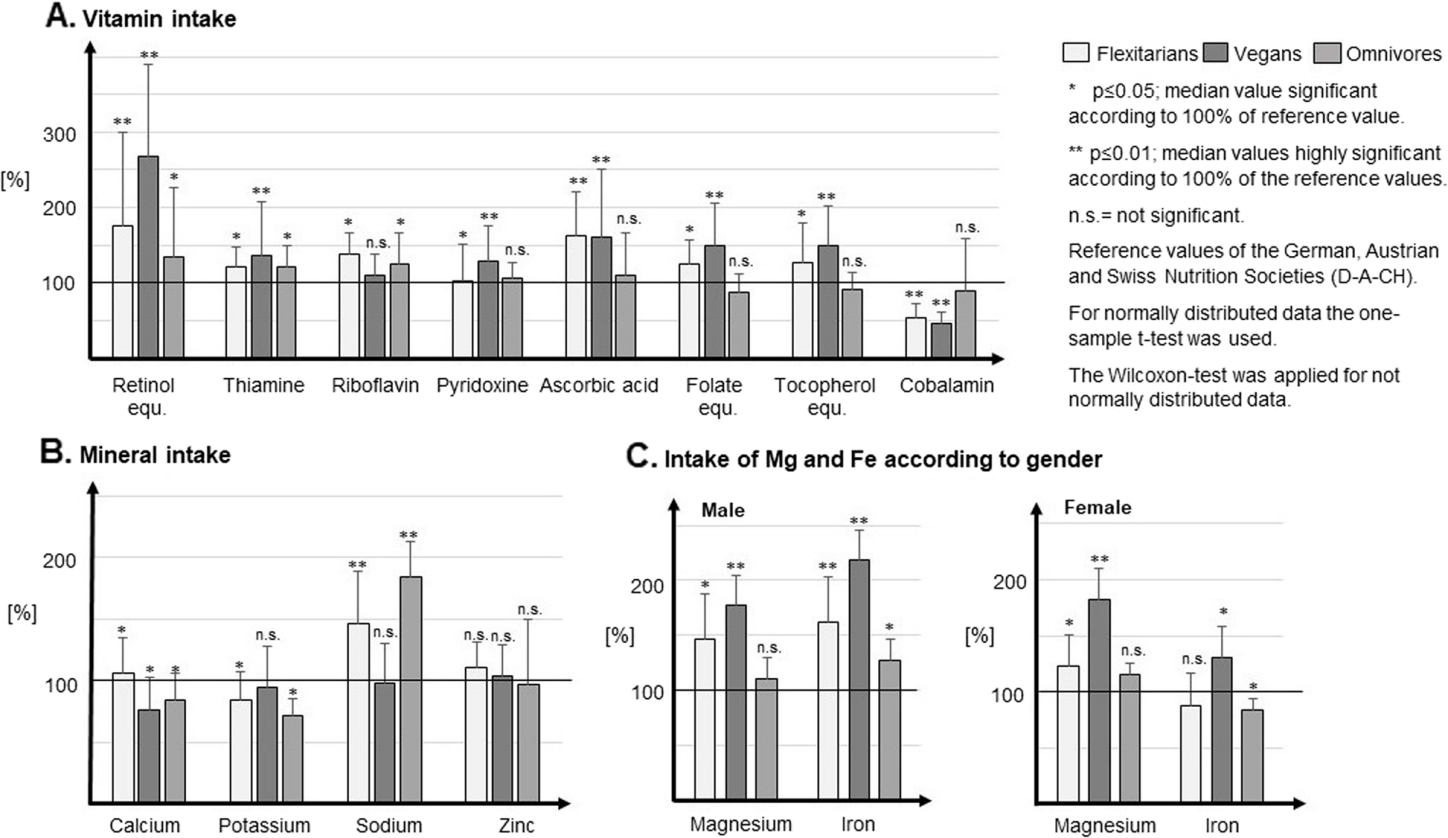
Micronutrient intake of vitamins and minerals based on 3-day dietary record according to the reference values [100%]

The D-A-CH DRIs for most micronutrients were met in all groups, although some micronutrients were consumed in significantly lower or higher amounts (Fig. 2A-C, Additional file 1). FXs significantly exceeded the DRI for ascorbic acid, thiamine, riboflavin, pyridoxine as well as folate, retinol and tocopherol equivalents. Similarly, high intakes were observed for Vs for thiamine, pyridoxine, ascorbic acid and folate, retinol and tocopherol equivalents. In contrast, none of the groups reached the DRI for cobalamin [4 µg], with Vs having the lowest cobalamin intake. The daily intake of dietary vitamin D (without supplements) was very low in the entire group with FX: 2.11 µg/d (1.20–3.21); V: 1.57 µg/d (0.85–3.33); and OMN: 1.94 µg/d (1.19–2.54). Vitamin D intakes were not compared with reference intakes because the study was conducted over several seasons with varying UV radiation and no single vitamin D intake recommendation could be used for comparison.

When comparing mineral intakes, FXs met the DRI for calcium [1000 mg], whereas, Vs and OMNs were below. Neither FXs nor OMNs met the recommendations for potassium [4000 mg], whereby Vs reached them. Moreover, FXs as well as OMNs exceeded the recommended values for sodium [1500 mg], while Vs showed an adequate intake. Regardless of diet and sex, zinc intake rates met recommendations. Additionally, in all three diets adequate intake for iron [10 mg] and magnesium [350 mg] was observed in men. In contrast, women met the DRI for magnesium [300 mg] in all groups, but only the V women met the DRI for iron [15 mg].

### Biochemical markers

#### Biomarkers of cobalamin and folate status

Based on the applied markers serum cobalamin, Holo-TC and 4cB_12_, the cobalamin status of most participants in all three groups was low but adequate (Table 3). Only 13% of FXs, 15% of Vs, and 14% of OMNs showed deficient cobalamin concentrations [< 150 pmol/L]. Based on 4cB_12_ 13% of FXs, 10% of OMNs, and 9% of Vs showed an undersupply in cobalamin [< -0.5 to -2.5]. However, serum cobalamin and Holo-TC concentrations were significantly lower in FXs than in Vs. Not surprisingly, comparison between SU and non-SU showed for all markers that the cobalamin status was worse in non-SU. The lowest concentrations in both markers for Vs who did not take supplements. Furthermore, the 4cB12 marker showed the least favourable cobalamin status for FXs. Despite the lowest values for FXs overall, the evaluation of Vs non-SU showed the lowest concentrations, significantly less than non-SU in OMNs and FXs. Notably, in contrast, SU in the V group showed the highest concentrations in cobalamin, Holo-TC and 4cB12 across the three diets.

**Table 3:** Biomarkers of cobalamin and folate status

**Parameter**	**FX** ***n* = 32**	***P*** **FX-V**	**V** ***n* = 33**	***P*** **V-OMN**	**OMN** ***n* = 29**	***P*** **FX-OMN**	***P*** **overall**
	${\varvec{n}}(\boldsymbol{\%})/\widetilde{{\varvec{x}}}({\varvec{I}}{\varvec{Q}}{\varvec{R}})$		${\varvec{n}}(\boldsymbol{\%})/\widetilde{{\varvec{x}}}({\varvec{I}}{\varvec{Q}}{\varvec{R}})$		${\varvec{n}}(\boldsymbol{\%})/\widetilde{{\varvec{x}}}({\varvec{I}}{\varvec{Q}}{\varvec{R}})$		
**Cobalamin [pmol/L]**	204 (169–244)	**0.010**	300 (200–428)	0.658	236 (198–306)	0.311	**0.013**^**a**^
Deficient [< 150 pmol/L], n (%)	4 (13)		5 (15)		4 (14)		0.609^b^
SU	218 (177–328)	n.s	318 (221–458)	n.s	235 (196–349)	n.s	0.138^a^
Non-SU	204 (168–232)	n.s	156 (111–236)	n.s	237 (198–306)	n.s	0.064^a^
**Holo-TC [pmol/L]**	60.9 (46.8–74.4)	**0.022**	94.2 (56.1–116)	1.000	76.6 (55.0–92.5)	0.162	**0.021**^**a**^
Deficient [< 35 pmol/L], n (%)	0 (0)		3 (9)		1 (3)		0.445^b^
SU	63.5 (44.7–72.3)	**0.035**	107 (66.3–118)	1.000	90.3 (65.9–129)	0.256	**0.038**^**a**^
Non-SU	59.5 (50.4–74.9)	0.158	47.5 (33.6–56.1)	**0.013**	74.9 (54.8–86.3)	0.465	**0.015**^**a**^
**MMA [nmol/L]**	224 (186–292)	n.s	207 (160–262)	n.s	219 (164–242)	n.s	0.352^a^
Elevated [> 271 nmol/L], n (%)	11 (34)		8 (24)		4 (14)		0.347^b^
**tHcy [µmol/L]**	12.0 (9.80–15.1)	n.s	9.70 (7.90–11.8)	n.s	10.5 (9.40–13.2)	n.s	0.095^a^
Elevated [> 10 µmol/L], n (%)	24 (75)		13 (39)		19 (66)		0.500^b^
**4cB**_**12**_	-0.07 (-0.35–0.19)	**0.006**	0.34 (-0.18–0.74)	0.633	0.20 (-0.10–0.31)	0.290	**0.009**^**a**^
Undersupply [< -0.5 to -2.5], n (%)	4 (13)		3 (9)		3 (10)		0.448^b^
Deficient [< -2.5], n (%)	0 (0)		0 (0)		0 (0)		
SU	0.19 (0.15–0.25)	n.s	0.46 (0.19–0.85)	n.s	0.22 (-0.04–0.53)	n.s	0.057^a^
Non-SU	-0.26 (-0.36–0.12)	0.620	-0.44 (-1.05–0.10)	**0.037**	0.17 (-0.11–0.23)	0.206	**0.028**^**a**^
**Folate [nmol/L]**	16.8 (10.6–22.0)	0.064	21.75 (15.1–35.1)	**0.033**	15.8 (9.52–21.0)	1.000	**0.019**^**a**^
Deficient [< 6.8 nmol/L], n (%)	1 (3)		0 (0)		2 (7)		0.157^b^
SU	21.6 (18.5–27.1)	n.s	23.1 (15.8–37.8)	n.s	19.7 (13.3–25.8)	n.s	0.621^a^
Non-SU	13.6 (10.1–19.9)	n.s	16.0 (10.6–20.3)	n.s	15.4 (9.29–21.0)	n.s	0.825^a^

*P* ≤ 0.05 shows statistical significance

*P*-values in bold represent statistical significance

*IQR* Interquartile range, *FX* Flexitarians, *V* Vegans, *OMN* Omnivores, *SU* Supplement user, *non-SU* Non-supplement user, *Holo-TC* Holotranscobalamin, *MMA* Methylmalonic acid, *tHcy* tTotal homocysteine, *4cB*_*12*_ Four markers combined cobalamin-indicator adjusted by age: probable cobalamin deficiency (< -2.5), possible cobalamin deficiency (-2.5 to -1.5), low cobalamin (-1.5 to -0.5), cobalamin adequacy (-0.5 to 1.5), elevated cobalamin (> 1.5) [27], Folate (serum): cut-off-values bases on [42]

^a^Kruskal-Wallis test with post hoc test and Bonferroni correction, ^b^Chi-Square test

Prevalence of elevated MMA [> 271 nmol/L] and tHcy [> 10 µmol/L] concentrations was highest in FXs, followed by OMNs and Vs. Median serum folate concentrations of all three diet groups showed a low but adequate folate supply: Only 3% of FXs, 0% of Vs, and 7% of OMNs were deficient [< 6.8 nmol/L]. As expected, Vs had significantly higher serum folate concentrations, followed by FXs and OMNs.

#### 25(OH)D and parathormone status

The median 25(OH)D concentrations in the FX group was the lowest at 46.6 nmol/L, while Vs and OMNs had slightly higher concentrations with 55.6 nmol/L and 59.6 nmol/L, respectively (Table 4). Consequently, Vs and OMNs were above the cut-off for an adequate vitamin D status [> 50 nmol 25(OH)D/L], while FXs were below the cut-off. As a result, the prevalence of an insufficient/deficient vitamin D status [< 49.9 nmol 25(OH)D/L] was highest in FXs (53%), followed by Vs (34%) and OMNs (27%). As expected, SU showed higher concentrations of 25(OH)D than non-SU across all diet groups. However, all differences were not significant. Vs had significantly higher PTH concentrations compared to FXs (*p* = 0.026) and OMNs (*p* = 0.015), however, median PTH concentrations in all three groups were within the reference range (15–65 pg/ml) [44–46].

**Table 4:** Vitamin D and parathormone (PTH) status

**Parameter**	**FX** ***n* = 32**	***P*** **FX-V**	**V** ***n* = 33**	***P*** **V-OMN**	**OMN** ***n* = 29**	***P*** **FX-OMN**	***P*** **overall**
	${\varvec{n}}(\boldsymbol{\%})/\widetilde{{\varvec{x}}}({\varvec{I}}{\varvec{Q}}{\varvec{R}})$		${\varvec{n}}(\boldsymbol{\%})/\widetilde{{\varvec{x}}}({\varvec{I}}{\varvec{Q}}{\varvec{R}})$		${\varvec{n}}(\boldsymbol{\%})/\widetilde{{\varvec{x}}}({\varvec{I}}{\varvec{Q}}{\varvec{R}})$		
**25(OH)D [nmol/L]**	46.6 (33.0–65.4)	n.s	55.6 (45.6–75.3)	n.s	59.6 (46.4–70.3)	n.s	0.172^a^
Desirable [≥ 75 nmol/L], n (%)	6 (19)	n.s	9 (27)	n.s	6 (21)	n.s	0.461^b^
Sufficient [50–74.9 nmol/L], n (%)	9 (28)	n.s	13 (39)	n.s	15 (52)	n.s	0.603^b^
Insufficient [25–49.9 nmol/L], n (%)	13 (40)	n.s	10 (30)	n.s	5 (17)	n.s	0.495^b^
Deficient [< 25 nmol/L], n (%)	4 (13)	n.s	1 (4)	n.s	3 (10)	n.s	0.313^b^
**25(OH)D [nmol/L]**							
SU	57.6 (37.9–78.6)	n.s	60.9 (46.1–83.1)	n.s	64.1 (51.1–67.8)	n.s	0.779^a^
Non-SU	44.8 (32.7–64.1)	n.s	48.8 (45.1–55.9)	n.s	56.2 (45.1–73.1)	n.s	0.360^c^
**PTH (pg/ml)**							
Total	31.5 (26.5–40.5)	**0.026**	36.9 (33.6–50.2)	**0.015**	30.6 (26.3–38.7)	1.000	**0.007**^**a**^
f	31.5 (28.4–42.5)	n.s	42.9 (33.6–50.7)	n.s	32.1 (29.1–38.7)	n.s	0.102^a^
m	31.9 (25.9–35.9)	n.s	36.6 (31–8-46.2)	n.s	28.3 (26.1–39.2)	n.s	0.074^c^

25-(OH)-D: Cut-off-values bases on [40]

*P* ≤ 0.05 shows statistical significance

*P*-values in bold represent statistical significance

*IQR* Interquartile range, *FX* Flexitarians, *V* Vegans, *OMN* Omnivores, *SU* Supplement user, *non-SU* Non-supplement user

^a^Kruskal-Wallis test with post hoc test and Bonferroni correction, ^b^Chi-Square test, ^c^One-way ANOVA

#### Biomarkers of iron status and haematological parameters

Based on markers serum iron and transferrin saturation, many people in this cohort have a poor iron status (Table 5). As expected, males had higher ferritin concentrations than females, with males in the OMN group having the highest ferritin concentrations, followed by males in the FX and V groups (*p* = 0.001). Women showed the same trend, but without significance. Women tend to be more affected by iron deficiency (ferritin < 15 µg/l) and iron insufficiency (transferrin saturation < 16%) than men. However, depleted iron stores (ferritin), anaemia (Hb) and iron deficiency anaemia (MCV) were observed only in a few subjects of the three dietary groups and mainly in women. In summary, the iron status of participants in the FX and V groups was the least favourable, whereas only a few women in the OMNs group had an inadequate iron status. It should be noted that no differences were observed between the three dietary groups for any of the iron status markers (except ferritin concentration in men).

**Table 5:** Biomarkers of iron status and haematological parameters

		**FX** ***n* = 32**	***P*** **FX-V**	**V** ***n* = 33**	***P*** **V-OMN**	**OMN** ***n* = 29**	***P*** **FX-OMN**	***P*** **overall**
		${\varvec{n}}(\boldsymbol{\%})/\widetilde{{\varvec{x}}}({\varvec{I}}{\varvec{Q}}{\varvec{R}})$		${\varvec{n}}(\boldsymbol{\%})/\widetilde{{\varvec{x}}}({\varvec{I}}{\varvec{Q}}{\varvec{R}})$		${\varvec{n}}(\boldsymbol{\%})/\widetilde{{\varvec{x}}}({\varvec{I}}{\varvec{Q}}{\varvec{R}})$		
**Gender, n (%)**	f	18 (56)		18 (55)		13 (45)		
	m	14 (44)		15 (45)		16 (55)		
**Parameter [unit]**								
**Iron serum [µmol/L]**	f	15.5 (10.0–23.0)	n.s	17.0 (12.9–21.0)	n.s	19.0 (15.0–20.0)	n.s	0.578^a^
	m	19.5 (14.0–22.0)	n.s	22.0 (16.0–24.0)	n.s	19.5 (18.0–21.5)	n.s	0.567^b^
Pre-latent iron deficiency [< 20-14 µmol/L], n (%)	f	5 (28)	n.s	5 (28)	n.s	4 (31)	n.s	0.692^c^
	m	3 (21)	n.s	5 (33)	n.s	7 (44)	n.s	0.848^c^
Deficient [< 14 µmol/L], n (%)	f	7 (39)	n.s	6 (33)	n.s	3 (23)	n.s	0.627^c^
	m	2 (14)	n.s	1 (7)	n.s	0 (0)	n.s	0.083^c^
**Ferritin [µg/l]**	f	51.5 (22.0–60.0)	n.s	32.0 (21.0–42.0)	n.s	53.0 (39.0–71.0)	n.s	0.077^b^
	m	92.5 (47.0–156)	1.000	75.0 (48.0–114)	**0.001**	172 (135–220)	**0.018**	**0.001**^**b**^
Depleted iron stores [< 15 µg/l], n (%)	f	1 (6)	n.s	2 (11)	n.s	1 (8)	n.s	0.238^c^
	m	0 (0)	-	0 (0)	-	0 (0)	-	-
**Transferrin saturation [%]**	f	11.5 (7.29–16.6)	n.s	13.5 (10.3–21.4)	n.s	14.6 (10.3–17.5)	n.s	0.645^a^
	m	17.2 (13.4–19.8)	n.s	19.4 (14.2–22.5)	n.s	16.3 (14.6–20.2)	n.s	0.485^b^
Insufficient iron supply [< 16%], n (%)	f	13 (72)	n.s	11 (61)	n.s	7 (54)	n.s	0.335^c^
	m	6 (43)	n.s	5 (33)	n.s	8 (50)	n.s	0.292^c^
**Hb [g/dl]**	f	13.5 (12.8 -13.7)	n.s	13.2 (12.5–13.8)	n.s	14.1 (13.1–14.4)	n.s	0.164^a^
	m	14.7 (14.0–15.3)	n.s	14.7 (14.4–15.4)	n.s	14.8 (14.6–15.3)	n.s	0.532^b^
Anaemia								
[< 12 g/dl], n (%)	f	1 (6)	-	1 (6)	-	0 (0)	-	^−^
[< 13 g/dl], n (%)	m	1 (7)	n.s	1 (7)	n.s	0 (0)	n.s	0.747^c^
**Hct [%]**	f	39.8 (38.3–40.5)	n.s	39.4 (37.5–41.2)	n.s	40.1 (39.4–42.2)	n.s	0.197^b^
	m	43.1 (40.8–44.4)	n.s	42.8 (42.2–44.9)	n.s	44.0 (42.6–45.3)	n.s	0.245^a^
Lowered								
[< 36%], n (%)	f	1 (6)	-	0 (0)	-	0 (0)	-	-
[< 39%], n (%)	m	2 (14)	n.s	1 (7)	n.s	0 (0)	n.s	0.223^c^
**MCV [fl]**	f	88.7 (83.9–90.0)	n.s	89.0 (87.0–92.1)	n.s	88.7 (86.0–90.9)	n.s	0.289^a^
	m	85.8 (83.4–87.1)	n.s	87.6 (86.7–90.5)	n.s	87.4 (85.8–90.8)	n.s	0.110^b^
Iron deficiency anaemia [< 80 fl], n (%)	f	1 (6)	-	0 (0)	-	0 (0)	-	-
	m	1 (7)	n.s	0 (0)	n.s	1 (8)	n.s	0.157^c^

*P* ≤ 0.05 shows statistical significance

*P*-values in bold represent statistical significance

*IQR* Interquartile range, *FX* Flexitarians, *V* Vegans, *OMN* Omnivores, *f* Female, *m* Male, *Hb* Haemoglobin, *Hct* Haematocrit, *MCV* Mean corpuscular volume

^a^One-way ANOVA, ^b^Kruskal-Wallis test with post hoc test and Bonferroni correction, ^c^Chi-Square test

## Discussion

Plant-based diets have gained much interest in recent years and the proportion of the population adopting a FX diet is steadily increasing. While vegetarianism and veganism have been evaluated in numerous studies and compared with omnivorous diets [12, 15, 47–52], knowledge about flexitarianism is still limited. Therefore, the aim of the present study was to investigate the intake and endogenous nutrient status of flexitarians compared to vegans and omnivores within a healthy adult study group in Germany. Most of the relevant studies have only used food records to assess macro- and micronutrient intakes, and rarely assessed state-of-the-art endogenous nutrient status markers as in the present study. Our study is therefore of a pilot nature.

In general, the entire study cohort can be classified as healthy and above average active with more than 1 h of physical activity per day in all groups. Among the FXs, 32% reported using dietary supplements, compared to 24% of the OMNs. These results are comparable to the average supplement use observed in other German study cohorts of OMN subjects [49]. In addition, 82% of the Vs reported using dietary supplements. While this may seem high, it is consistent with the results of other studies in which V participants also reported supplement use between 75 and 90% [49, 51, 53, 54]. Thus, both the activity rates and the widespread use of supplements suggest that a proportion of this study cohort leads an active lifestyle and appears to be aware of the potential risks associated with their dietary choices.

All three groups were slightly below the recommended total energy intake with no relevant differences between the diets. However, protein requirements were met in all three groups. The desirable carbohydrate intake was practically only achieved by the Vs, while carbohydrate intake in FXs was halfway between the Vs and the OMNs. A higher fat and protein intake in OMNs compared to Vs in the present study was not surprising, and has been reported previously [12, 17, 19, 48]. The high fiber intake of FXs (and Vs) were close to the recommended amounts and can be considered beneficial [55].

The participants in the FX group reached the DRI for most vitamins (thiamine, riboflavin, pyridoxine, ascorbic acid as well as folate, retinol, and tocopherol equivalents) and minerals (zinc, calcium, magnesium). However, intake rates of FXs were even higher in the majority compared to OMNs. This can be attributed to the increased intake of plant foods, which are considered good sources for these micronutrients.

As green vegetables, fruits and legumes are important sources of folate, it was expected that Vs would have the highest serum folate concentrations compared to FXs and OMNs. This is in line with previous observations [12], where higher folate concentrations in Vs compared to meat eaters were described. In the present study, only one FX woman and two OMN men had deficient folate concentrations [< 6.8 nmol/l] [56]. A significantly higher prevalence of folate deficiency was found in OMNs in comparable studies [57, 58]. As low folate concentrations are also associated with contraceptive use [59], this could not be confirmed by the present data. Overall, the folate status showed that the prevalence of folic acid deficiency was low and can be considered non-critical in the present study population.

Given that the contribution of plant foods to cobalamin is negligible, the low cobalamin status in Vs, who did not take supplements, is not surprising and has been frequently observed in previous studies [12, 47–49, 60–63]. Although cobalamin intake of FXs (and Vs) was less than 50% of the DRI, the markers Holo-TC and 4cB_12_ showed that only a few participants across all diet groups showed cobalamin deficiency, in contrast to serum cobalamin. Holo-TC and the combined marker 4cB_12_ are considered more valid for assessing the long-term biostatus of cobalamin status [64–66], as cobalamin in serum can fluctuate daily and may inadequately represent the cobalamin status in tissues [67–69]. The body’s own cobalamin stores can last for several years. Most subjects eating a FX diet (59%) switched from an omnivore diet only a few years ago (< 5 years). However, the prevalence of cobalamin deficiency, as measured by 4cB_12_, was highest in the FXs compared to the Vs and OMNs. This also explains the highest prevalence of elevated MMA and tHcy in FXs. We expected that FX participants, most of whom have been on a low meat diet for several years, would still have adequate cobalamin stores. In conclusion, our data suggest that most FX participants have depleted cobalamin stores. Another explanation could be that the FX participants have been restricting their meat intake for a longer time. In consequence, FX subjects appear to be unaware of the critical supply of cobalamin, as only a few participants reported taking supplements. Therefore, to avoid cobalamin deficiency, FXs (similar to Vs) should consider cobalamin supplementation early, as is generally recommended for a V diet.

The most common sources of calcium are milk and dairy products. The finding that FXs had a higher calcium intake than Vs was therefore expected. An adequate intake of calcium, which is particularly important for bone health, is of major concern for Vs. Similarly, lower intakes of Vs compared to OMNs have already been reported [12, 15, 49, 52]. In fact, serum calcium is strictly homeostatically regulated and not directly related to dietary intake. Hence, it is not a suitable valid indicator of calcium status [40]. Therefore, we also examined PTH concentrations. Elevated PTH concentrations may indicate an increased risk of osteoporosis. Vs showed the highest PTH concentrations, but still within reference values. However, these findings emphasize the need for Vs to choose alternative, non-animal sources of calcium (e.g. mineral water, legumes) and/or appropriate supplementation [70, 71]. It should also be noted that the presence of oxalic acid reduces absorption [72].

Adequate vitamin D status is also of particular importance for calcium metabolism, bone health, cardiovascular diseases, immune system, and cancer prevention [73–75]. Dietary sources usually cover up only 10% to 20% of the vitamin D requirement and therefore do not significantly influence the vitamin D status [76]. The main part of vitamin D requirements must be met by a) endogenous synthesis of the vitamin requiring sunlight exposure [73, 77], and b) the supplementation of the vitamin or fortification of foods.

As expected and found in numerous studies [17, 77–79], intake of vitamin D from diet without supplements was very low in all groups (FX: 2.11 µg (1.20–3.21), V: 1.57 µg (0.85–3.33); OMN: 1.94 (1.19–2.54). Since the study was conducted at different seasons of the year (March to August 2020) resulting in individually varying vitamin D requirements, no uniform DRI could be assumed. We therefore did not compare the vitamin D intake with the DRI. In the absence of endogenous synthesis, vitamin D intake recommendation is between 15 µg/d [80] and 20 µg/d by [28, 81].

In the present study, the median 25(OH)D concentration in serum was poorest in the FXs compared to Vs and OMNs. In the FXs, the prevalence of an insufficient and deficient vitamin D status was 53% compared to 34% in the Vs and 27% in the OMNs. As dietary vitamin D intake via food did not differ between the groups, the difference in vitamin D status is due to other causes. Rather, the observed 25(OH)D concentrations are strongly influenced by the intake of supplements. For the FXs and Vs, the median 25(OH)D concentrations were higher for the SU than for the non-SU, while the median 25(OH)D concentrations of the non-SU were below 50 nmol/L and thus indicate an insufficient or deficient vitamin D status.

Another reason for the differences in the vitamin D status between the three groups could be differences in self-synthesis depending on sunlight (UV-B) exposure. Intensity of UV-B radiation during the study and individual sunlight exposure of the participants are unknown. However, if we infer “duration of sunlight exposure” from “physical activity” - assuming that sports are essentially done outdoors - FXs were almost as active as Vs and significantly more active than OMNs. Nevertheless, it is not possible to explain the differences in the vitamin D status between the three groups. However, our results clearly indicate that the risk for vitamin D deficiency increases without using supplements.

For iron, men’s dietary intakes were above the DRI in all groups. In women, however, iron intakes were below the DRI for FXs and OMNs, while female Vs showed iron intakes above the DRI. Similar results with significantly higher iron intakes in vegetarians or Vs compared to OMNs have already been found in other studies [12, 49]. The reason for this could be a high consumption of iron-rich plant foods such as whole grains or legumes. However, the availability of plant iron species is significantly lower compared to haem-bound iron from meat and meat products. The simultaneous intake of food components that reduce (e.g., phytate) or increase (e.g., ascorbic acid) iron availability also plays an important role and reduces the significance of purely quantitative iron intake values. A conclusion on the iron supply can therefore only be drawn employing biomarkers of iron status and haematological parameters.

As expected, all iron status markers showed significantly better iron status in men than in women in all groups. The ferritin concentrations clearly show that the OMNs had the highest iron stores despite lower iron intake, which is primarily related to the higher availability of haem-bound iron from meat and meat products as described above. Despite the highest absolute iron intake, the Vs showed the lowest ferritin status. However, serum iron and transferrin saturation values indicate that insufficient iron intake and resulting iron deficiency occur not only in women, but also in men of all three groups. It should be noted that the number of people with an inadequate iron status in each group was very small. In consequence, the results should be treated with caution and cannot be generalised.

### Limitations

The study has several potential limitations. The lack of statistical significance (*p* > 0.05) between groups for some variables (e.g., 25(OH)D, serum iron) may be due to the relatively small sample size and power of the study. This is to be expected given the exploratory nature of the study and justifies the need for a future study with larger sample sizes that would be well powered to detect small effect sizes and/or more conclusively indicate whether or not certain measures differ between diets. As a FX diet is not clearly defined, our results cannot be extrapolated to a plant-based, meat-reduced FX diet in general. Most participants were recruited through notice boards and online communities dedicated to plant-based diets. Therefore, a particular health consciousness of some subjects cannot be excluded. However, this is a general phenomenon in nutrition studies. The assessment of nutrient intake via 3-day dietary records may be somewhat biased due to possible over- or underreporting by participants and the fact that the diet during the three days may not be representative of the subject’s usual diet. In addition, the estimation of nutrient amounts in foods and food products using software-based calculation tools is vulnerable to potential errors in nutrient composition found in food databases of the nutrient intake calculation software. For a more precise evaluation of predictors of the vitamin D status, the daily sunlight exposure and the corresponding season should have been recorded in more detail. Also, we did not accurately document the intake of supplements. The study participants only stated whether they used dietary supplements, but no detailed information on frequency, composition, and dosage of dietary supplements was collected. Finally, we have no information on the prevalence of helicobacter pylori infection or atrophic gastritis in our cohort to predict dietary malabsorption of cobalamin.

## Conclusion

In summary, the results showed that all three diets were able to provide adequate intakes of most macro- and micronutrients. However, differences could be observed in certain aspects: FXs had higher intakes of fiber, retinol equ., ascorbic acid, folate equ., tocopherol equ., calcium, and magnesium compared to OMNs. Conversely, biomarker analysis revealed a prevalence of cobalamin and iron deficiencies for FXs. Furthermore, all three groups had a very low dietary vitamin D intake and a high prevalence of an insufficient/deficient vitamin D status. Remarkably, the Vs showed awareness of micronutrient deficiencies, with over 80% using supplements. In contrast, FXs appeared to be less aware of such deficiencies, with only about 30% using supplements. In the case of vitamin D in particular, the risk of a deficiency increases if supplements are not used, regardless of the diet.

### Supplementary Information

**Additional file 1.** Daily intake of vitamins and minerals.

## Data Availability

The datasets used and/or analyzed during the current study available from the corresponding author on reasonable request.
