# Supplementary material for: Nutritional status of flexitarians compared to vegans and omnivores - a cross-sectional pilot study
Source: BMC Nutr. 2023 Nov 28;9:140. doi: 10.1186/s40795-023-00799-6 (PMC10685640; doi:10.1186/s40795-023-00799-6)
Supplement: Supplementary file 1 — Additional file 1. Daily intake of vitamins and minerals. [file 40795_2023_799_MOESM1_ESM.docx]

**Nutritional Status of Flexitarians Compared to Vegans and Omnivores - a Cross-Sectional Pilot Study**

Anja Bruns, Josefine Nebl, Wiebke Jonas, Andreas Hahn, Jan Philipp Schuchardt

Institute of Food Science and Human Nutrition, Leibniz University Hannover, Hannover, Germany

**Supplementary Data**

Additional file 1 Daily intake of vitamins and minerals.

**Additional file 1 Daily intake of vitamins and minerals.**

| **Parameter**[unit] | **FX (n=32)**  f=18, m=14 | **p-value FX-V**  **+** | **V (n=33)**  f=18, m=15 | **p-value**  **V-OMN**  **++** | **OMN (n=29)**  f=13, m=16 | **p-value**  **FX-OMN**  **+++** | **p-value overall** | **D-A-CH intake**  **recommendations^1^** |
| --- | --- | --- | --- | --- | --- | --- | --- | --- |
|  | $n/\tilde{x}(IQR)$ |  | $n/\tilde{x}(IQR)$ |  | $n/\tilde{x}(IQR)$ |  |  |  |
| **Vitamins** | | | | | | | | |
| Retinol equivalent [µg]  f  total  m | 1477 (1113-2126)  1309 (1055-2126)  1097 (924-1873) | n.s.  0.110  0.121 | 1817 (1283-2888)  1948 (1329-2888)  2318 (1354-2961) | n.s.  **0.001**  **0.006** | 1393 (715-1614)  1027 (715-1628)  994 (696-1694) | n.s.  0.287  1.000 | 0.108^b^  **0.001^b^**  **0.007^b^** | 700µg    850µg |
| Thiamine [mg]  f  total  m | 1.21 (1.05-1.45)  1.29 (1.05-1.54)  1.45 (1.05-2.01) | n.s.  n.s.  0.200 | 1.35 (1.28-1.61)  1.48 (1.31-2.26)  1.95 (1.34-2.82) | n.s.  n.s.  **0.011** | 1.39 (1.22-1.61)  1.27 (1.10-1.61)  1.44 (1.07-1.94) | n.s.  n.s.  1.000 | 0.353^b^  0.058^b^  **0.013^b^** | 1.0mg    1.2mg |
| Riboflavin [mg]  f  total  m | 1.50 (1.20-1.70)  1.58 (1.26-1.80)  1.68 (1.32-2.00) | n.s.  n.s.  n.s. | 1.21 (1.01-1.53)  1.22 (0.94-1.53)  1.22 (0.93-1.73) | n.s.  n.s.  n.s. | 1.67 (1.25-1.84)  1.27 (1.10-1.61)  1.44 (1.07-1.94) | n.s.  n.s.  n.s. | 0.186^a^  0.148^a^  0.247^a^ | 1.1mg    1.4mg |
| Pyridoxine [mg]  f  total  m | 1.44 (1.24-2.11)  1.51 (1.24-2.17)  2.05 (1.38-2.42) | n.s.  0.085  0.166 | 1.65 (1.38-2.44)  2.05 (1.54-2.76)  2.43 (1.65-3.31) | n.s.  0.093  **0.027** | 1.54 (1.43-1.78)  1.56 (1.31-1.95)  1.59 (1.31-2.23) | n.s.  1.000  1.000 | 0.461^b^  0.051^b^  **0.025^b^** | 1.4mg    1.6mg |
| Biotin [µg]  f  total  m | 55.0 (42.7-60.6)  55.0 (42.7-62.7)  52.7 (48.6-75.5) | n.s.  n.s.  0.694 | 43.9 (34.2-54.4)  51.2 (37.7-72.5)  72.3 (43.6-92.9) | n.s.  n.s.  **0.010** | 46.6 (31.7-61.3)  43.2 (31.7-57.5)  41.5 (31.0-54.3) | n.s.  n.s.  0.328 | 0.359^b^  0.127^b^  **0.013^b^** | 40µg |
| Cobalamin [µg]  f  total  m | 2.15 (1.45-3.24)  2.12 (1.44-2.90)  2.05 (1.38-2.76) | 0.811  **0.001**  1.000 | 1.65 (1.29-2.44)  1.81 (1.49-2.45)  2.05 (1.61-2.76) | **0.002**  **<0.001**.**0.032** | 3.60 (2.52-7.42)  3.70 (2.26-6.33)  3.98 (2.08-5.10) | 0.051  **0.002**  **0.042** | **0.003^b^**  **<0.001^b^**  **0.014^b^** | 4µg |
| Ascorbic acid [mg]  f  total  m | 131 (90.0-210)  159 (100-210)  184 (136-206) | n.s.  1.000  n.s. | 164 (135-297)  161 (135-276)  159 (119-276) | n.s.  **0.017**  n.s. | 109 (82.4-158)  109 (65.1-164)  112 (54.5-192) | n.s.  0.161  n.s. | 0.111^b^  **0.018^b^**  0.088^b^ | 95mg    110mg |
| Vitamin D [µg]  f  total  m | 2.61 (1.20-3.65)  2.11 (1.20-3.21)  1.86 (1.27-2.57) | n.s.  n.s.  n.s. | 1.60 (0.78-3.77)  1.57 (0.85-3.33)  1.57 (0.88-2.95) | n.s.  n.s.  n.s. | 1.76 (1.26-2.29)  1.94 (1.19-2.54)  1.99 (0.96-2.57) | n.s.  n.s.  n.s. | 0.622^b^  0.374^b^  0.675^b^ | 15-20µg^2^ |
| Tocopherol equivalent [mg]  f  total  m | 14.7 (8.89-20.9)  15.9 (9.32-21.6)  17.7 (12.9-25.3) | n.s.  0.247  1.000 | 18.5 (12.8-30.6)  19.4 (12.8-27.7)  19.7 (12.7-27.7) | n.s.  **0.001**  **0.009** | 11.3 (10.8-15.4)  11.3 (9.14-15.6)  10.6 (7.05-15.7) | n.s.  0.167  0.140 | 0.093^b^  **0.001^b^**  **0.009^b^** | 12mg    14mg |
| Vitamin K [µg]  f  total  m | 118 (92.2-212)  115 (72.1-212)  114 (54.7-211) | 0.947  0.173  0.251 | 181 (97.4-454)  161 (118-358)  153 (139-323) | **0.001**  **0.000**  **0.001** | 49.8 (34.4-99.9)  51.6 (34.1-97.4)  69.1 (32.8-92.6) | **0.025**  **0.003**  0.195 | **0.001^b^**  **0.001^b^**  **0.001^b^** | 60µg  70µg |
| Folate equivalent [µg]  f  total  m | 354 (297-398)  397 (303-470)  383 (371-547) | 0.362  0.233  1.000 | 415 (315-545)  448 (360-616)  504 (360-639) | **0.037**  **0.000**  **0.001** | 275 (240-331)  265 (222-336)  243 (211-339) | 0.839  **0.027**  **0.021** | **0.039^b^**  **0.001^b^**  **0.001^b^** | 300µg |
| Niacin equivalent [mg]  f  total  m | 21.5 (19.2-27.2)  23.0 (19.2-31.4)  23.8 (20.5-33.2) | n.s.  1.000  n.s. | 20.2 (13.8-23.3)  21.0 (17.0-26.9)  26.6 (17.0-30.0) | n.s.  **0.007**  n.s. | 28.1 (20.3-32.0)  29.1 (24.4-37.2)  31.6 (24.9-39.8) | n.s.  0.111  n.s. | 0.112^b^  **0.008^b^**  0.248^b^ | 12mg    15mg |
| Pantothenic acid [mg]  f  total  m | 4.56 (3.73-5.68)  4.81 (3.73-6.36)  5.80 (4.68-7.49) | n.s.  n.s.  n.s. | 4.25 (3.31-6.10)  5.21 (3.78-6.81)  5.75 (4.16-6.94) | n.s.  n.s.  n.s. | 4.34 (3.60-5.08)  4.17 (3.55-6.12)  4.00 (3.48-7.28) | n.s.  n.s.  n.s. | 0.928^b^  0.537^b^  0.248^b^ | 5mg |
| **Minerals** | | | | | | | | |
| Calcium [mg]  f  total  m | 988 (814-1154)  1062 (953-1344)  1241 (1062-1415) | **n.s.**  **0.001**  **0.009** | 744 (599-937)  757 (599-1032)  796 (596-1203) | n.s.  1.000  1.000 | 929 (678-1056)  838 (728-1056)  833 (728-1058) | **n.s.**  **0.009**  **0.002** | 0.117^a^  **0.001^b^**  **0.002^a^** | 1000mg |
| Potassium [mg]  f  total  m | 3010 (2671-3730)  3352 (2671-4279)  3988 (3053-4990) | n.s.  0.139  0.565 | 3770 (2897-4961)  3771 (3089-5118)  4497 (3241-5436) | n.s.  **0.000**  **0.004** | 2993 (2434-3167)  2847 (2378-3418)  2759 (2327-3836) | n.s.  0.212  0.184 | 0.070^b^  **0.001^b^**  **0.006^a^** | 4000mg |
| Magnesium [mg]  f  total  m | 399 (314-469)  401 (331-532)  512 (363-656) | **0.021**  **0.006**  0.271 | 583 (410-718)  604 (427-733)  618 (427-912) | **0.000**  **0.000**  **0.003** | 346 (298-375)  347 (299-405)  387 (308-450) | 0.382  0.164  0.473 | **0.001^b^**  **0.001^b^**  **0.005^b^** | 300mg    350mg |
| Zinc [mg]  f  total  m | 9.33 (8.6-10.5)  9.84 (8.84-13.0)  12.3 (9.71-15.5) | n.s.  n.s.  n.s. | 10.1 (8.29-13.4)  10.4 (8.29-13.5)  11.9 (8.10-14.4) | **n.s.**  **n.s.**  **n.s.** | 12.3 (8.59-13.3)  10.7 (8.84-13.5)  10.2 (8.99-14.0) | n.s.  n.s.  n.s. | 0.449^b^  0.937^b^  0.557^b^ | 8mg^3^    14mg^3^ |
| Iron [mg]  f  total  m | 13.2 (12.0-15.7)  13.4 (11.7-16.3)  16.1 (11.6-19.6) | 0.054  **0.009**  0.201 | 19.6 (14.8-22.9)  21.5 (14.8-24.4)  21.8 (14.1-26.4) | **0.009**  **0.000**  **0.000** | 12.6 (11.3-14.0)  12.6 (9.92-14.0)  12.7 (8.84-13.9) | 1.000  0.277  0.211 | **0.007^b^**  **0.001^b^**  **0.001^b^** | 10mg    15mg |
| Sodium [mg]  f  total  m | 2032 (1561-2622)  2201 (1624-2828)  2577 (2201-2828) | 0.061  **0.003**  0.059 | 1339 (986-1762)  1463 (1004-1959)  1537 (1006-2712) | **0.001**  **0.001**  **0.002** | 2767 (2414-3217)  2767 (2349-3198)  2703 (2278-3196) | **0.030**  **0.019**  0.815 | **0.001^a^**  **0.001^a^**  **0.002^a^** | 1500mg |

IQR= Interquartile range, EN%=percent of total energy, FX=Flexitarians, V=Vegans, OMN=Omnivors

+ p-value FX-V: significant or not-significant values between flexitarians and vegans

++ p-value V-OMN: significant or not-significant values vegans and omnivores

+++ p-value FX-OMN: significant or not-significant values between flexitarians and omnivores

1 D-A-CH

2 dependents on season of the year, UV/sunlight radiation, skin veiling

3 with medium phytate supply

a for normally distributed data, the One-Way-Anova (p≤0.05) was applied

b for not normally distributed data, the Kruskal-Wallis-Test (p≤0.05) with Post/hoc Bonferroni correction was used

p>0.05 was considered significant

p-values in bold represent statistical significance
